# VX765, a Specific Caspase-1 Inhibitor, Alleviates Lung Ischemia Reperfusion Injury by Suppressing Endothelial Pyroptosis and Barrier Dysfunction

**DOI:** 10.1155/2021/4525988

**Published:** 2021-12-22

**Authors:** Siyi Wu, Zhao Li, Mengling Ye, Chunxia Liu, Hao Liu, Xiaojing He, Yi Qin, Fangte Liang, Linghui Pan, Fei Lin

**Affiliations:** ^1^Department of Anesthesiology, Guangxi Medical University Cancer Hospital, Nanning, 530021 Guangxi Zhuang Autonomous Region, China; ^2^Key Laboratory for Basic Science and Prevention of Perioperative Organ Dysfunction, Guangxi Medical University Cancer Hospital, Nanning, 530021 Guangxi Zhuang Autonomous Region, China; ^3^Department of Experimental Research, Guangxi Medical University Cancer Hospital, Nanning, 530021 Guangxi Zhuang Autonomous Region, China

## Abstract

Lung ischemia reperfusion injury (LIRI) is a complex pathophysiological process with high morbidity and mortality. An important pathophysiological characteristic of LIRI is endothelial barrier dysfunction, although the mechanism involved in this process remains unclear. VX765, a specific caspase-1 inhibitor, has been shown to have a protective effect against several diseases including sepsis, atherosclerosis, and glial inflammatory disease. The objective of this study was to determine whether VX765 had a protective effect in LIRI. The results showed that lung ischemia/reperfusion (I/R) and oxygen/glucose deprivation and reoxygenation (OGD/R) induced endothelial pyroptosis and barrier dysfunction characterized by an inflammatory response. Treatment with VX765 successfully alleviated I/R- and OGD/R-induced endothelial pyroptosis and barrier dysfunction by inhibiting caspase-1 *in vivo* and *in vitro*. In conclusion, these findings showed that VX765 provided effective protection against lung I/R-induced endothelial pyroptosis and barrier dysfunction.

## 1. Introduction

Lung ischemia reperfusion injury (LIRI) leads to lung tissue damage during blood supply after a period of ischemia [1]. LIRI is therefore a major complication after lung transplantation and pulmonary embolism [2, 3] and causes organ dysfunction and worse outcomes in patients [4, 5]. Studies have indicated that endothelial barrier dysfunction is a key factor in ischemia/reperfusion (I/R) injury [6]. The findings of our previous study were consistent with these other studies as we showed that lung I/R released large amounts of inflammatory factors and induced endothelial barrier dysfunction [7]. Recent studies also demonstrated that pyroptosis plays a key role in I/R injury [8–10], although it remains unclear whether pyroptosis is related to LIRI-induced endothelial barrier dysfunction.

Pyroptosis is a special type of programmed cell death and is distinguished from apoptosis and necroptosis by its characteristic features of cell swelling, cell membrane rupture, cell lysis, and a marked proinflammatory response [11]. The canonical pyroptosis pathway is dependent on caspase-1 and cleaved gasdermin D (GSDMD) forming cell membrane pores that allow the exchange of ions [12, 13]. During the process of pyroptosis, several cellular events occur following activation by caspases such as caspase-1/4/5/11 and include cleaving of GSDMD and secretion of inflammatory factors, such as interleukin-1*β* (IL-1*β*) and interleukin-6 (IL-6) [14–16]. VX765 is a newly developed and selective small-molecule caspase-1 inflammation inhibitor, which reduces inflammation *in vitro* and *in vivo*. There is evidence that VX765 treatment protects against traumatic brain injury by attenuating pyroptosis [17]. However, the underlying role of VX765 in LIRI and whether it inhibits pyroptosis remain largely unknown. The objectives of this study were to determine the specific role of VX765 in LIRI and to investigate the potential effects of VX765 to attenuate pyroptosis and endothelial barrier dysfunction in LIRI.

## 2. Materials and Methods

### 2.1. Animals and Experimental Protocol

6–8-week-old male C57BL/6J mice were obtained from the Experimental Animal Center of the Guangxi Medical University (Nanning, China) and maintained under specific pathogen-free conditions. The mice were divided randomly into control, I/R, and I/R+VX765 groups (*n* = 15 per group). Mice in the I/R+VX765 groups received an intraperitoneal injection of 30 mg/kg VX765 on 3 consecutive days before ischemia and 30 min before reperfusion. The lung I/R *in vivo* model was established as reported previously [18]. Briefly, the mice in the I/R and I/R+VX765 groups were 50 mg/kg anaesthetized with pentobarbital and the left hilum then clamped for 1 h, followed by reperfusion for 6 h. The left lung was collected for follow-up experiments after modeling. All the animal experimental protocols were conducted with the approval of the Institutional Animal Care and Use Committee of Guangxi Medical University (Nanning, China; Approval No. 202103015, Animal's Studies Ethic Date: March 2021–March 2023).

### 2.2. Cell Culture and Cellular Model of OGD/R

Pulmonary microvascular endothelial cells (PMVECs) were obtained from Fine Test, Wuhan, China, and then cultured at 37°C in a humidified atmosphere in 5% CO_2_ in 89% Dulbecco's modified Eagle medium (DMEM, Thermo Fisher, Wuhan, China) supplemented with 10% fetal bovine serum (Gibco, Carlsbad, CA, USA), 0.5% penicillin, and 0.5% streptomycin The cells were divided into control, oxygen/glucose deprivation and reoxygenation (OGD/R), and OGD/R+VX765 groups. The pulmonary microvascular endothelial cells (PMVECs) were cultured for 1 h in an atmosphere of 1% O_2_, 5% CO_2_, and 94% N_2_, followed by culture for 6 h at 37°C in glucose-containing medium in an atmosphere of 5% CO_2_ and 95% O_2_ as previously reported [19]. The cells in the OGD/R+VX765 group had 10 *μΜ* VX765 added 1 h before reoxygenation. The cells were then collected for follow-up experiments after modeling.

### 2.3. Reagents and Antibodies

VX765 was purchased from Jizhi Biochemical Technology (Shanghai, China); GSDMD and caspase-1 antibodies from ABclonal Company (Wuhan, China); CD31 from Servicebio (Wuhan, China); *β*-actin from Cell Signaling Technology (Danvers, MA, USA); fluorophore-labeled goat anti-rabbit secondary antibody (Alexa Fluor 488, 594) and goat anti-rabbit immunoglobulin horseradish peroxidase (IgG-HRP) and anti-mouse IgG-HRP, bicinchoninic acid assay (BCA) protein detection kits from Beyotime (Shanghai, China); real-time qPCR (RT-qPCR) kits from Takara (Tokyo, Japan); lactate dehydrogenase (LDH) kits from BestBio (Shanghai, China); ZO-1 and VE-cadherin antibodies from Abcam (Cambridge, UK); and IL-6, tumor necrosis factor-*α* (TNF-), and IL-1*β* enzyme-linked immunosorbent assay (ELISA) kits from Elabscience Biotechnology (Wuhan, China).

### 2.4. Transmission Electron Microscopy

The lung tissue samples were cut into approximately 1 mm pieces and fixed in 3% glutaraldehyde for 24 h, followed by 1% osmic acid for 1–2 h. The tissue samples were dehydrated in graded concentrations of acetone before embedding in resin and then cut into ultrathin sections with an ultramicrotome and observed with a transmission electron microscope (H-7560, Tokyo, Japan).

### 2.5. Hematoxylin Eosin Staining

The lung tissue specimens were fixed in 4% paraformaldehyde and then embedded in paraffin for sectioning. The sections were stained with hematoxylin and eosin (H&E) and photographed under a light microscope (EVOS FL AutoLife Technologies). Lung injury was scored as previously described [20].

### 2.6. Real-Time qPCR

Total RNA was extracted from lung tissue or cells using Trizol (catalog no.15596-026, Invitrogen) according to the manufacturer's instructions. Total RNA (1 *μ*g) was used as a template in a 20 *μ*L reverse transcription reaction using the PrimeScript™ RT reagent kit (Takara, Japan) according to the manufacturer's instructions. The cDNA was amplified using the following primers: *β*-actin forward, 5′-GGCTGTATTCCCCTCCATCG-3; *β*-actin reverse, 5′-CCAGTTGGTAACAATGCCATGT-3′; IL-1*β* forward, 5′-ATGAGAGCATCCAGCTTCAA-3′, IL-1*β* reverse, 5′-TGAAGGAAAAGAAGGTGCTC; IL-6 forward, 5′-TAGTCCTTCCTACCCCAATTTCC-3, IL-6 reverse, 5′-TTGGTCCTTAGCCACTCCTTC-3; TNF-*α* forward, 5′-CCCTCACACTCAGATCATCTTCT-3, TNF-*α* reverse, 5′-GCTACGACGTGGGCTACAG-3′; Casp-1 forward, 5′-ACAAGGCACGGGACCTATG-3′, Casp-1 reverse, 5′-TCCCAGTCAGTCCTGGAAATG-3; GSDMD forward, 5′-CCATCGGCCTTTGAGAAAGTG-3′, GSDMD reverse, 5′-ACACATGAATAACGGGGTTTCC-3′. Relative gene expression was quantified relative to the expression of *β*-actin using the 2^−*ΔΔ*ct^ method.

### 2.7. Enzyme-Linked Immunosorbent Assay (ELISA)

The concentrations of IL-6, TNF-*α*, and IL-1*β* in lung tissues, mouse serum, or cell culture supernatant were quantified using ELISA kits according to the manufacturer's instructions.

### 2.8. Cell Viability

A cell counting kit-8 (CCK-8 kit) was used to detect cell viability using the same cell culture conditions as described above. The cells in the different groups were counted and the concentration adjusted to 1 × 10^4^/mL, followed by inoculation in 96-well plates at 100 *μ*L/well. The cells were seeded in triplicate for each treatment group. The 96-well plates were placed in an incubator (37°C and 5% CO_2_), incubated for an appropriate time, followed by the addition of 10 *μ*L of CCK-8 solution to each well and further incubation for 1 h. A microplate reader was used to detect the absorbance at 450 nm. Blank wells (medium and CCK) and control wells (untreated cells, medium and CCK) were also tested. Cell survival rate was calculated as (optical density (OD) value of the experimental group − OD value of the blank group)/(OD value of the control group − the blank group).

### 2.9. Immunofluorescence Staining

The lung tissue or PMVECs were fixed with 4% paraformaldehyde for 1 h, washed 3 times with phosphate-buffered saline (PBS), permeabilized with 0.1% Triton X-100 for 20 min, and blocked with 5% bovine serum albumin for 30-60 min. The samples were incubated with either ZO-1 (1 : 200), VE-cadherin (1 : 200), caspase-1 (1 : 100), GSDMD (1 : 100), or CD31 (1 : 100) antibodies in a humidified box overnight at 4°C and then washed 3 times with PBST (5 min each time), followed by staining with fluorophore-conjugated secondary antibody (Alexa Fluor 488 or 546) and further incubation for 1 h at room temperature in the dark. 4′,6-Diamidino-2-phenylindole (DAPI) was used for nuclear staining. After washing with PBST, the staining pattern was observed using a fluorescence microscope (Nikon Eclipse C1, Nikon).

### 2.10. Western Blotting

The protein concentration of extracts of lung tissue homogenates or PMVECs was measured using a BCA protein assay kit (P0011, Beyotime). The same amount of protein was applied to a 10% SDS polyacrylamide gel for electrophoresis and then transferred to a polyvinylidene fluoride (PVDF) membrane. The membrane was blocked with TBST containing 5% milk for 1 h and then incubated at 4°C overnight with caspase-1 (1 : 1000), GSDMD (1 : 1000), *β*-actin (1 : 5000), and primary antibody and anti-rabbit IgG-HRP (1 : 1000) or anti-mouse IgG-HRP (1 : 1000) secondary antibody. The protein bands were identified by an enhanced detection chemiluminescence (ECL) system.

### 2.11. LDH Release

A lactic dehydrogenase (LDH) cytotoxicity detection kit was used to evaluate the level of LDH in the culture supernatant, according to the manufacturer's instructions. The medium in each cell sample wells was aspirated for testing in a new 96-well culture plate II, followed by the addition of 100 *μ*L LDH working solution to the original cell culture plate I, gentle shaking at room temperature for 30-45 min, and then addition of 10 *μ*L LDH reaction solution to cultures plates I and II. The LDH release rate was calculated as OD II/(OD II − OD I)∗100%. The experiment was repeated more than three times.

### 2.12. Statistical Analysis

SPSS software (IBM, USA) was used for the data analyses and the data plotted using GraphPad Prism (GraphPad Software, USA). The quantitative data were expressed as mean ± standard error of the mean (SEM) and compared using Tukey's test and analysis of variance (ANOVA). Differences with a *P* value < 0.05 were considered statistically significant.

## 3. Results

### 3.1. I/R-Induced Lung Injury and Inflammation in the Mouse Model and OGD/R-Induced Inflammation and Endothelial Barrier Dysfunction in the Endothelium Model

**Figure 1:**
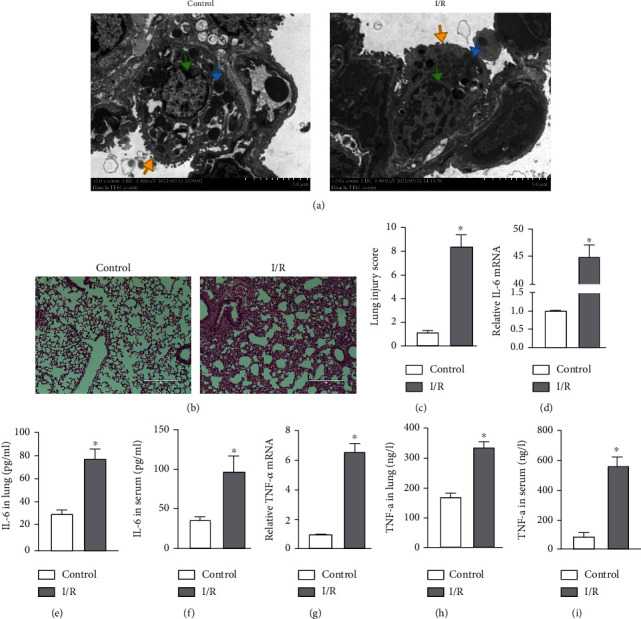
Lung I/R induced inflammatory response and tissue injury. (a–i) Mouse lung tissue and serum collected from the control and I/R groups for the following experiments. (a) Transmission electron microscopy to evaluate ultrastructural changes: microvilli (yellow arrow), mitochondria (blue arrow), and nucleus (green arrow). Scale bar, 5 *μ*m. (b) Evaluation of pathology by H&E staining. Scale bar, 100 *μ*m. (c) Lung injury score based on H&E images. (d) IL-6 mRNA levels measured by RT-qPCR. (e, f) IL-6 levels in the lung and serum measured by ELISA. (g) TNF-*α* mRNA levels measured by RT-qPCR. (h, i) TNF-*α* level in the lung and serum measured by ELISA. The data are expressed as mean ± SEM (*n* = 7 per group). ^∗^*P* < 0.05 vs. the control group.

To demonstrate that I/R caused lung damage *in vivo*, we constructed a mouse model of lung I/R. The ultrastructural changes in the lung in the I/R group observed by transmission electron microscopy, compared with those in the control group, showed nuclear swelling, a reduction in cell surface microvilli, and mitochondrial damage in alveolar type II cells (Figure 1(a)). Histological images and the injury score showed that I/R also induced severe lung injury (Figures 1(b) and 1(c)). Consistent with the fact that I/R injury is a pathological event in tissue injury and is characterized by an excessive inflammatory response [6], we showed increased levels of inflammatory factors including IL6 and TNF-*α* in lung tissue and serum. The mRNA and protein levels of IL-6 and TNF-*α* in lung tissue and serum were significantly higher in the I/R group compared with those measured in the control group (Figures 1(d)–1(i)). Taken together, these results showed that I/R caused lung damage and inflammation.

**Figure 2:**
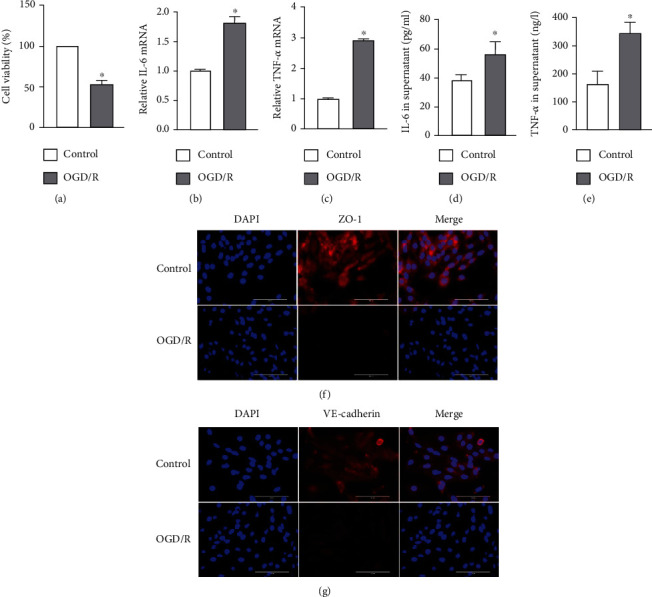
OGD/R-induced endothelial barrier disruption and cell injury. (a–g) PMVECs and supernatant were collected from the control and OGD/R groups for the following experiments. (a) Viability of PMVECs assessed by CCK-8. (b, c) IL-6 and TNF-*α* mRNA levels measured by RT-qPCR. (d, e) IL-6 and TNF-*α* protein levels in cell supernatant measured by ELISA. (f, g) Immunofluorescence micrographs of PMVEC ZO-1 and VE-cadherin (red) identified by immunofluorescence. DAPI was used to stain the cell nuclei. Scale bar, 200 *μ*m. The data are expressed as mean ± SEM. Each experiment was performed independently at least 3 times. ^∗^*P* < 0.05 vs. the control group.

We constructed an OGD/R cell model to mimic I/R *in vitro*. Consistent with our previous findings, the mRNA and protein levels of IL-6 and TNF-*α* were significantly higher in the OGD/R group (Figures 2(b)–2(e)). We also assessed the effect of OGD/R on vascular barrier function by detecting tight junction proteins, including ZO-1 and VE-cadherin. The cellular distribution and expression of ZO-1 and VE-cadherin were reduced after OGD/R (Figures 2(f) and 2(g)). As shown in Figure 2(a), the viability of PMVECs was significantly decreased in the OGD/R group. These results demonstrated that OGD/R caused endothelial barrier dysfunction and a decline in cell viability.

### 3.2. Lung I/R Induced by Endothelial Pyroptosis *In Vivo* and *In Vitro*

**Figure 3:**
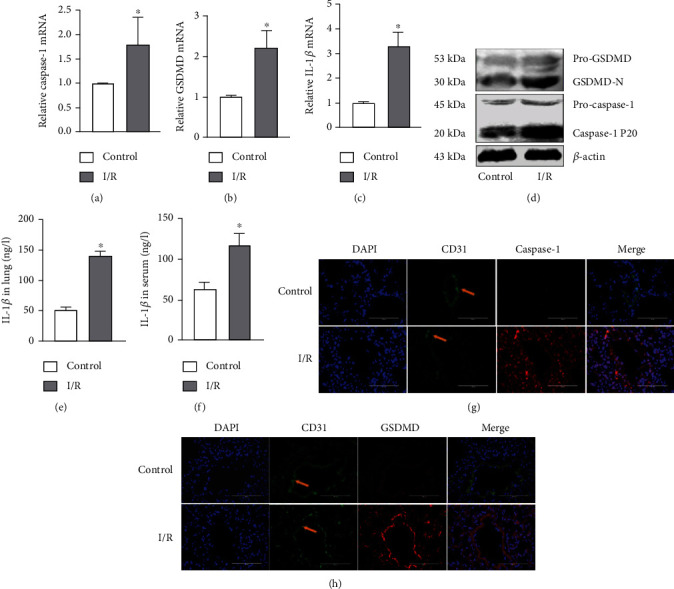
I/R induced endothelial pyroptosis in vivo. (a–h) Mouse lung tissue and serum were collected from the control and I/R groups for the following experiments. (a–c) Caspase-1, GSDMD, and IL-1*β* mRNA levels measured by RT-qPCR. (d) Protein levels of pro-GSDMD, GSDMD-N, pro-caspase-1, and caspase-1 P20 by detected by Western blotting. (e, f) IL-1*β* protein level in lung tissue and serum measured by ELISA. (g, h) Costaining of CD31 (green) with caspase-1 or GSDMD (red) and the vascular endothelium (yellow arrow). DAPI was used to stain the cell nuclei. Scale bar, 200 *μ*m. The data are expressed as mean ± SEM (*n* = 7 per group). ^∗^*P* < 0.05 vs. control.

To demonstrate that endothelial pyroptosis is involved in lung I/R, we examined the expression and localization of pyroptosis markers in lung tissues. Caspase-1, GSDMD, and IL-1*β* mRNA levels were markedly higher in the I/R group than in the control group (Figures 3(a)–3(c)). Compared with the control group, lung I/R increased the expression of caspase-1 p20 and GSDMD-N, while the expression of pro-GSDMD and pro-caspase-1 showed no change (Figure 3(d)). The IL-1*β* levels in lung tissue and serum were also increased in the I/R group (Figures 3(e) and 3(f)). We detected localization of pyroptosis *in vivo* using double immunostaining of the endothelium marker (CD31) and caspase-1 or GSDMD. The staining pattern showing CD31 costained with caspase-1 and GSDMD confirmed that lung I/R caused endothelial pyroptosis. Similar changes in caspase-1 and GSDMD expression were observed by Western blotting. Moreover, the expression of CD31 was decreased in the I/R group (Figures 3(g) and 3(h)). These results confirmed that endothelial pyroptosis was involved in lung I/R.

**Figure 4:**
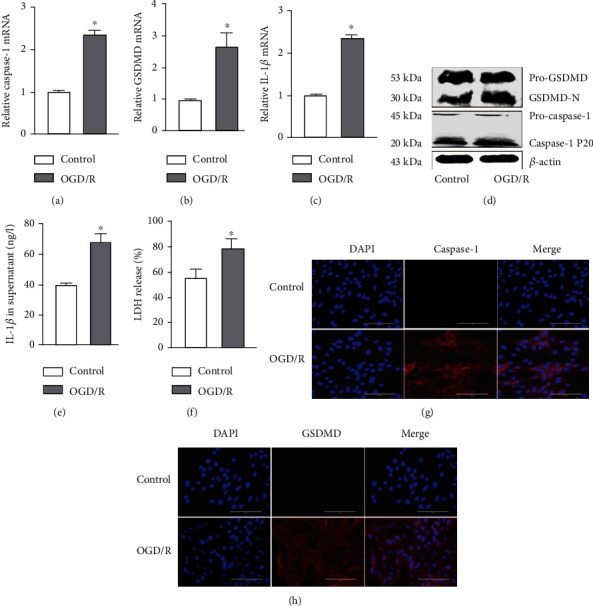
OGD/R-induced endothelial pyroptosis in vitro. (a–h) PMVECs and supernatant were collected from the control and OGD/R groups for the following experiments. (a–c) Caspase-1, GSDMD, and IL-1*β* mRNA levels measured by RT-qPCR. (d) Protein levels of pro-GSDMD, GSDMD-N, pro-caspase-1, and caspase-1 P20 detected by Western blotting. (e) Protein level of IL-1*β* in the cell supernatant measured by ELISA. (f) LDH release rate. (g, h) Immunofluorescence staining for caspase-1 or GSDMD (red). DAPI was used to stain the cell nuclei. Scale bar, 200 *μ*m. The data are expressed as mean ± SEM. Each experiment was performed independently at least 3 times. ^∗^*P* < 0.05 vs. control.

To further examine whether endothelial pyroptosis was related to OGD/R *in vitro*, as indicated in Figures 4(a)–4(c), caspase-1, GSDMD, and IL-1*β* mRNA levels in PMVECs were shown to be increased in the OGD/R group compared with the control group (Figures 4(a)–4(c)). OGD/R increased the expression of caspase-1p20 and GSDMD-N in the OGD/R group compared with the control group, whereas the expression of pro-GSDMD and pro-caspase-1 did not change (Figure 4(d)). In addition, IL-1*β* protein levels in the cell supernatants were increased (Figure 4(e)). Because the damaged cell membrane in pyroptotic cells can lead to the release of LDH [21], we performed a LDH release assay. The assay showed that PMVECs in the OGD/R group had a significant increase in the release of LDH into the cultural supernatant compared with that observed in the control group (Figure 4(f)). The immunofluorescence results also showed increased protein expression of caspase-1 and GSDMD in the OGD/R group (Figures 4(g) and 4(h)). These findings suggested that I/R and OGD/R are significantly causal factors in endothelial pyroptosis.

### 3.3. VX765 Alleviated I/R-Induced Lung Injury and Inflammation and OGD/R-Induced Inflammation and Endothelial Barrier Dysfunction

**Figure 5:**
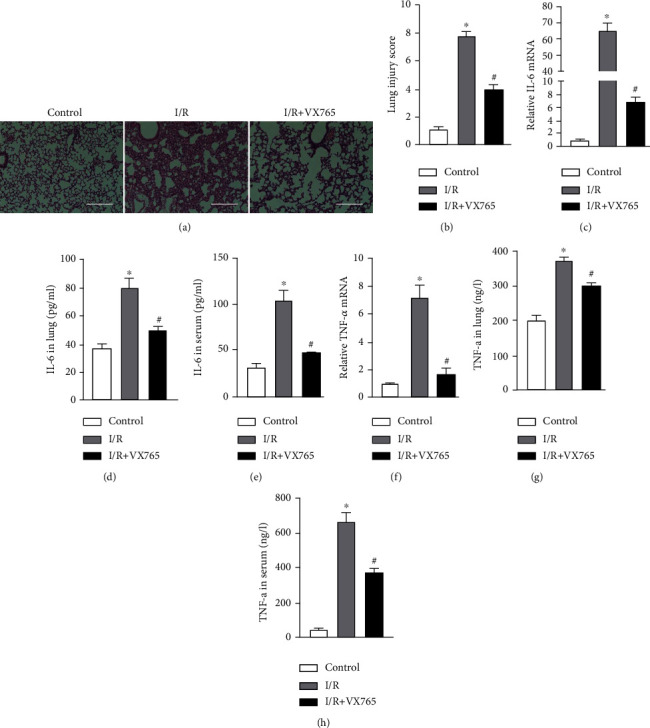
The effects of caspase-1 inhibitor VX765 on lung injury and inflammation. (a–h) Mouse lung tissue and serum were collected from each group for the following experiments. (a) The pathology score was evaluated by H&E staining. Scale bar, 100 *μ*m. (b) Lung damage was evaluated based on H&E images. (c) IL-6 mRNA levels measured by RT-qPCR. (d, e) IL-6 protein levels measured in lung and serum by ELISA. (f) TNF-*α* mRNA level measured by RT-qPCR. (g, h) TNF-*α* protein level in lung and serum measured by ELISA. The data are expressed as mean ± SEM (*n* = 7 per group). VX765 = 30 mg/kg. ^∗^*P* < 0.05 vs. control. ^#^*P* < 0.05 vs. I/R.

A previous study showed that VX765 inhibited lung injury and inflammation [22]. To confirm this role of VX765, a caspase-1 inhibitor, on the degree of injury and inflammation induced by I/R, we performed an experiment in a mouse model of lung I/R. Consistent with previous results, histopathology showed that lung injury was severe in the I/R group and was alleviated by VX765 (Figures 5(a) and 5(b)). VX765 also decreased the lung tissue and serum IL-6 mRNA and protein levels induced by I/R (Figures 5(c)–5(e)). Similar results were obtained for TNF-*α* (Figures 5(f)–5(h)). Taken together, these results showed that lung I/R markedly upregulated injury and inflammation; both of which were inhibited by VX765.

**Figure 6:**
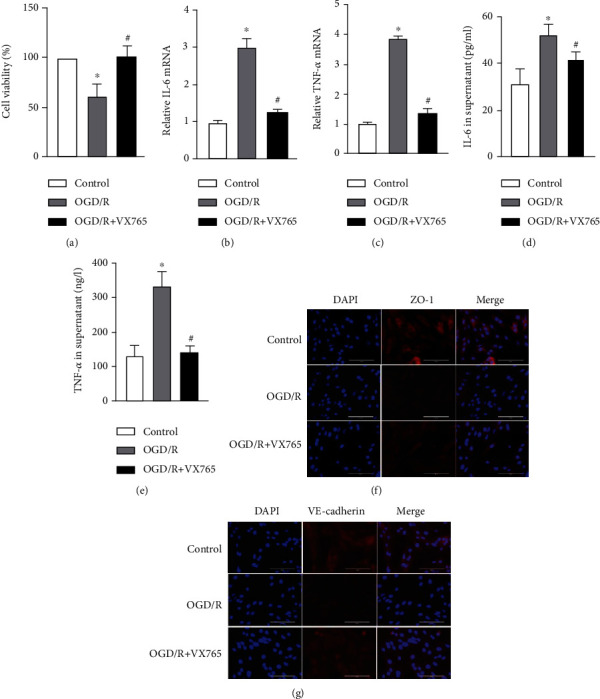
VX765 treatment attenuated injury and endothelial barrier function in vitro. (a–g) PMVECs and supernatant were collected from each group for the following experiments. (a) Viability of PMVECs assessed by CCK-8. (b, c) IL-6 and TNF-*α* mRNA levels measured by RT-qPCR. (d, e) IL-6 and TNF-*α* protein levels in the cell supernatants measured by ELISA. (f, g) Immunofluorescence micrographs of PMVEC ZO-1 (red) and VE-cadherin (red). DAPI was used to stain the cell nuclei. Scale bar, 200 *μ*m. The data are expressed as mean ± SEM. Each experiment was performed independently at least 3 times. VX765 = 10 *μΜ*. ^∗^*P* < 0.05 vs. control. ^#^*P* < 0.05 vs. OGD/R.

To further investigate the function of VX765 *in vitro*, we used VX765 in an experimental OGD/R cell model. This showed that mRNA and protein levels of IL-6 and TNF-*α* were lower in the VX765+OGD/R group compared with those in the OGD/R group (Figures 6(b)–6(e)). The results of immunofluorescence showed that VX765 improved the endothelial barrier function that had been damaged by OGD/R (Figures 6(f) and 6(g)). There is evidence that VX765 also upregulates cell viability [23]. We therefore examined PMVEC activity that showed cell viability was increased markedly by VX765 compared with that observed in the OGD/R group (Figure 6(a)). These results demonstrated that VX765 protects against inflammation and endothelial barrier dysfunction.

### 3.4. VX765 Attenuated Endothelial Pyroptosis Induced by Lung I/R and OGD/R

**Figure 7:**
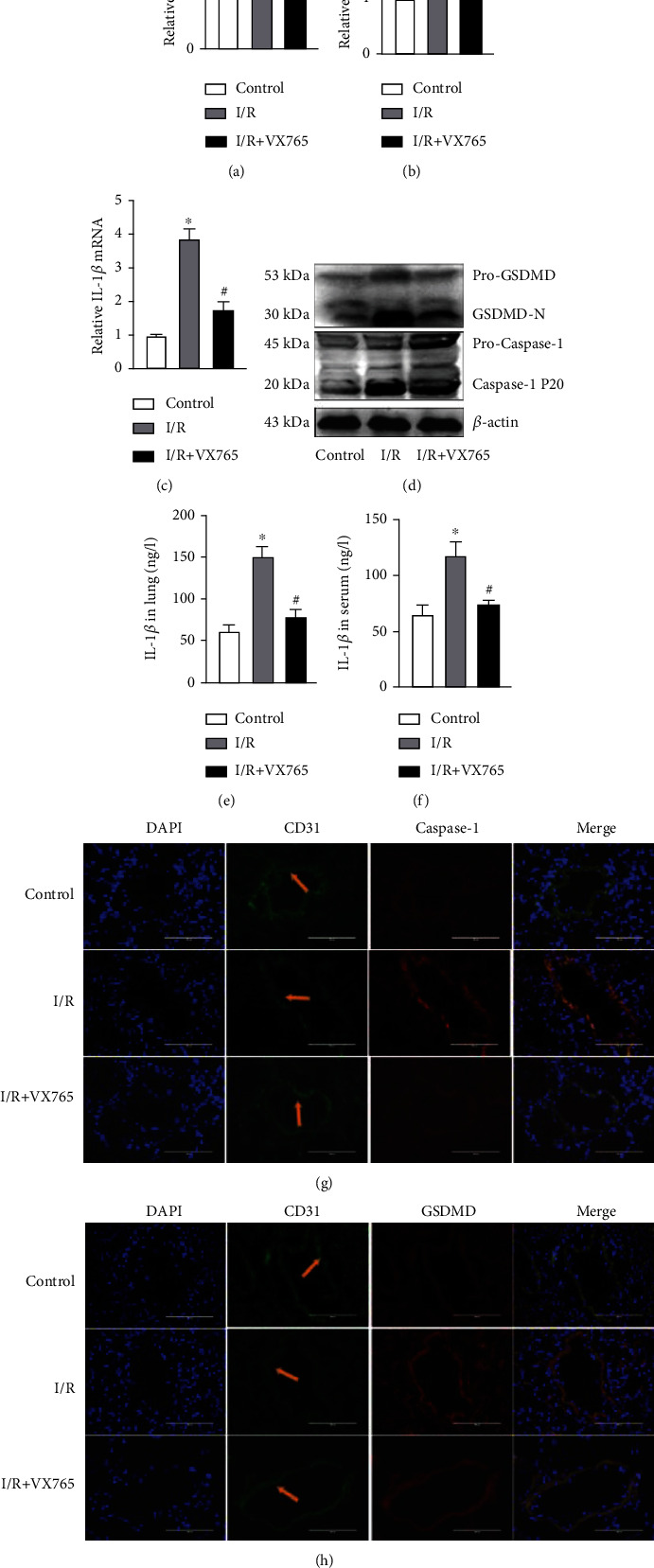
VX765 treatment attenuated I/R-induced endothelial pyroptosis. (a–h) Mouse lung tissue and serum were collected from each group for the following experiments. (a–c) Caspase-1, GSDMD, and IL-1*β* mRNA levels measured by RT-qPCR. (d) Protein levels of pro-GSDMD, GSDMD-N, pro-caspase-1, and caspase-1 P20 detected by Western blotting. (e, f) IL-1*β* protein level in lung tissue and serum measured by ELISA. (g, h) Colabeled CD31 (green) with caspase-1 or GSDMD (red). DAPI was used to stain the cell nuclei by immunofluorescence; vascular endothelium (yellow arrow), scale bar, 200 *μ*m. The data are expressed as mean ± SEM (*n* = 7 per group). VX765 = 30 mg/kg. ^∗^*P* < 0.05 vs. control. ^#^*P* < 0.05 vs. I/R.

The canonical pyroptosis pathway depends on caspase-1 [12]. We investigated the effect of VX765, a caspase-1 inhibitor, on endothelial pyroptosis induced by lung I/R. We observed that VX765 treatment decreased the mRNA expression levels of caspase-1, GSDMD, and IL-1*β* induced by I/R (Figures 7(a)–7(c)). I/R-induced expression of GSDMD-N and caspase-1 P20 was also decreased by VX765 treatment (Figure 7(d)). VX765 also consistently decreased lung tissue and serum IL-1*β* protein levels induced by I/R (Figures 7(e) and 7(f)). Immunofluorescence staining showed that VX765 increased the expression of CD31, while decreasing the expression of caspase-1 or GSDMD compared with the expression levels seen in the I/R group (Figures 7(g) and 7(h)). These results suggested that inhibition of caspase-1 protected against endothelial pyroptosis *in vivo*.

**Figure 8:**
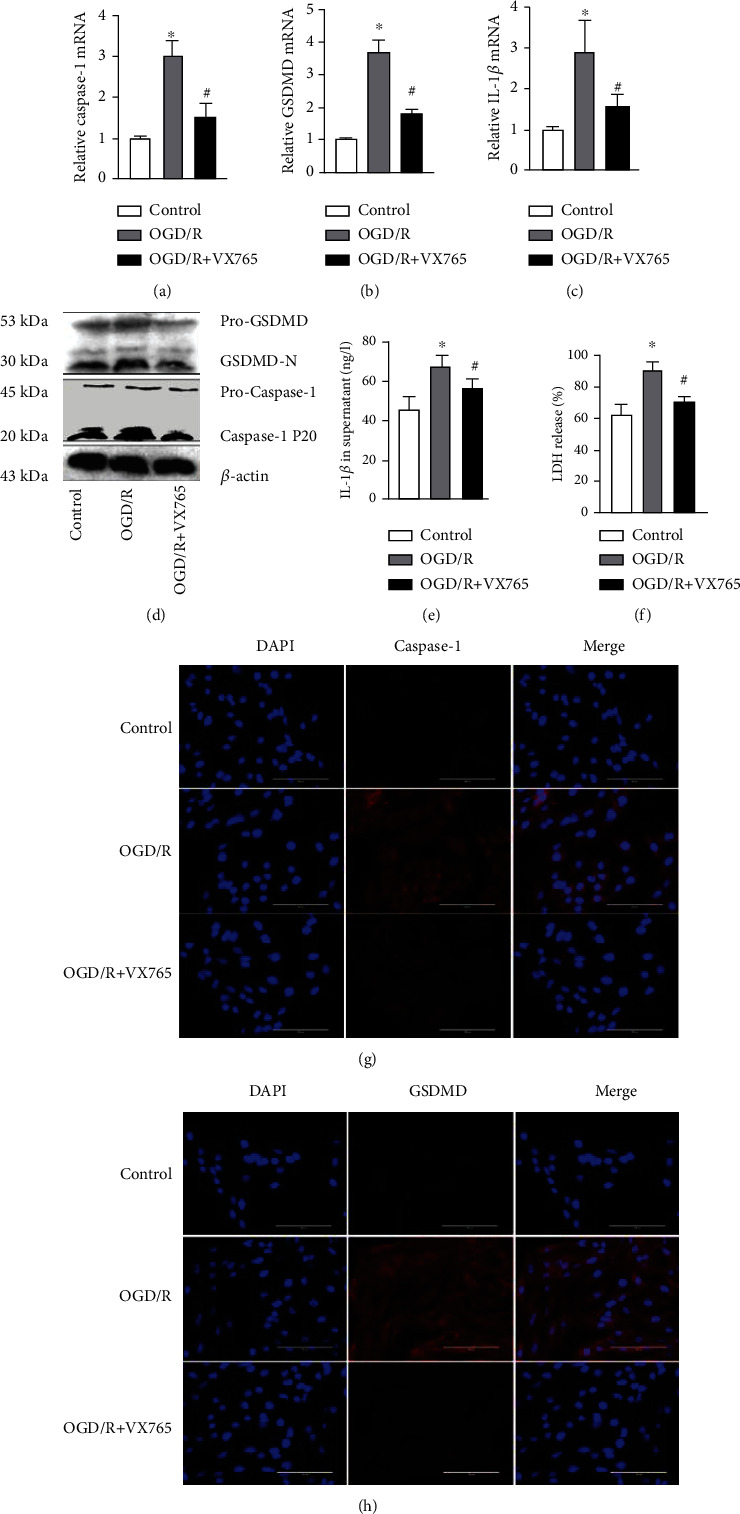
Effects of VX765 on PMVEC pyroptosis. (a–h) PMVECs and supernatant were collected from the controls and OGD/R group for the following experiments. (a–c) Caspase-1, GSDMD, and IL-1*β* mRNA levels. (d) Protein levels of pro-GSDMD, GSDMD-N, pro-caspase-1, and caspase-1 P20 measured by WB. (e) The protein level of IL-1*β* in the cell supernatant. (f) LDH release rate. (g, h) Immunofluorescence staining for caspase-1 or GSDMD (red). DAPI was used to stain the cell nuclei. Scale bar, 200 *μ*m. The data are expressed as mean ± SEM. Each experiment was performed independently at least 3 times. VX765 = 10 *μΜ*. ^∗^*P* < 0.05 vs. control. ^#^*P* < 0.05 vs. OGD/R.

To gain insights into our results from the *in vivo model*, we carried out further experiments in PMVECs that investigated the effect of VX765 on PMVEC pyroptosis by detecting the expression of caspase-1, GSDMD, and IL-1*β*. Consistent with the *in vivo* results, the OGD/R group showed obvious pyroptosis, with VX765 decreasing the mRNA levels of caspase-1, GSDMD, and IL-1*β* induced by OGD/R (Figures 8(a)–8(c)). In addition, the OGD/R-induced decrease in GSDMD-N and caspase-1P 20 was restored by VX765 in the PMVECs (Figure 8(d)), as well as the levels of IL-1*β* in lung tissue and serum (Figure 8(e)). VX765 also increased the expression of CD31 and decreased the expression of caspase-1 and GSDMD compared with that observed in the OGD/R group (Figures 8(f) and 8(g)). These results supported our *in vivo* findings that OGR/D induced endothelial pyroptosis and that VX765 mitigated this effect.

## 4. Discussion

Lung I/R induces acute lung injury or even the acute respiratory distress syndrome. Previous studies have shown that I/R may induce injury in different organs [24–26]. Consistent with these findings, the present study detected lung I/R-induced tissue injury and inflammation. We found that mice subjected to lung I/R exhibited severe pathological changes in lung morphology and ultrastructure as well as inflammation driven by IL-6 and TNF-*α* (Figure 1). These findings were consistent with our previous research [27]. Lung I/R has been shown to be related with endothelium function [6] that acts to form a barrier on the surface of the luminal blood vessels to separate the blood from the surrounding tissues. Endothelial barrier function therefore plays a central role in human health and diseases [28]. Our results confirmed the findings of our previous research that I/R induced dysfunction of endothelial tight junction proteins [19]. PMVECs subjected to OGD/R strongly upregulated inflammatory factors and disrupted endothelial barrier dysfunction (Figure 2), indicating that we had established models of I/R and OGD/R. The I/R model represented upregulation of lung injury and inflammation, while the OGD/R model represented upregulation of inflammation and endothelial barrier dysfunction.

**Figure 9:**
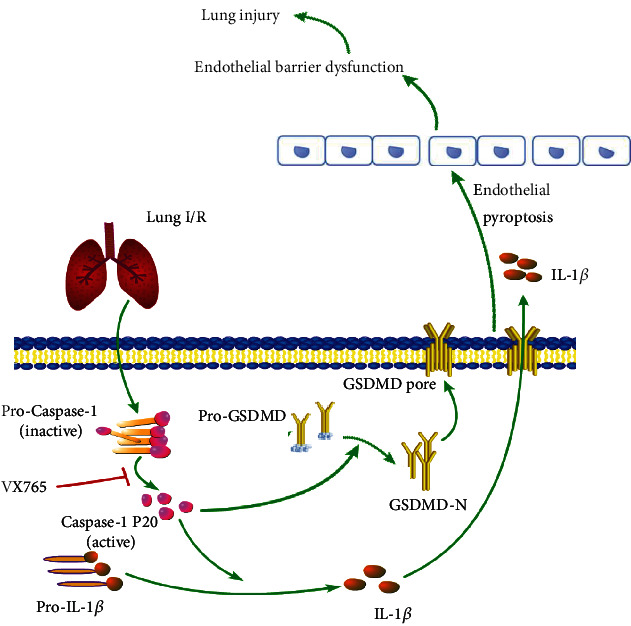
Schematic model of the role of VX765 in protecting against lung injury. Lung I/R activates caspase-1 P20 inducing GSDMD-N to form membrane pores, with IL-1*β* entering the cell through this pore. Endothelial pyroptosis causes barrier dysfunction leading to lung injury. However, VX765 can alleviate these phenomena.

Pyroptosis is a form of programmed cell death initiated by inflammasomes and is related closely with many diseases such as atherosclerosis, diabetic nephropathy, and cancer [21, 29, 30]. Intestinal I/R has been reported to cause intestinal barrier disruption and cell pyroptosis rather than directly affecting the endothelium [31]. In contrast, endothelial pyroptosis aggravates endotoxemia-induced lung injury [32], and therefore, prevention and control of endothelial pyroptosis are very important. These observations suggest that pyroptosis is activated and accompanied by an inflammatory response in acute lung injury. We demonstrated that I/R *in vivo* and OGD/R *in vitro* both increased the expression of GSDMD-N, which may be driven by caspase-1 P20. GSDMD-N oligomerizes to form large pores in the membrane that cause swelling and membrane rupture, along with the release of IL-1*β* [33] (Figure 9). Moreover, we showed that I/R upregulated the release of IL-1*β* and LDH. Taken together, these results confirm that I/R and OGD/R induced increased endothelial pyroptosis *in vivo* and *in vitro* (Figures 3 and 4).

Recent studies have reported that a highly selective caspase-1 inhibitor, VX765, further reduced the release of inflammatory cytokines and tissue damage during I/R [34, 35]. Consistent with this observation, we showed that VX765 attenuated lung pathological injury and reduced the release of IL-6 and TNF-*α*. There is evidence that VX765 counteracts the damage to the blood-brain barrier [36], and therefore, we investigated the potential therapeutic effect of VX765 against I/R in mice and OGD/R in PMVEC. These experiments showed that VX765 also regulated tight junctions that maintained the endothelial barrier and that ZO-1 and VE-cadherin helped to maintain the integrity of the endothelial barrier. Our study indicated that we had established *in vivo* and *in vitro* models of inhibited caspase-1 mitigated injury and improved endothelial barrier function. Our results indicated that VX765 contributed to repaired inflammation and endothelial barrier dysfunction during lung I/R and may therefore be a potential therapeutic target (Figures 5 and 6).

A previous study reported that VX765 alleviated inflammasome-induced pyroptosis by blocking caspase-1 signaling [37]. We therefore examined whether VX765 alleviated endothelial pyroptosis in I/R. Our investigations showed that I/R and OGD/R increased the expression of endothelial pyroptosis, whereas VX765 reduced the expression of GSDMD-N induced by caspase-1 P20 and decreased the release of IL-1*β* and LDH. These data demonstrated that I/R and OGD/R activated pyroptosis, which was, at least, partially mediated by GSDMD-N. VX765 inhibited the expression of GSDMD-N and IL-1*β* by inhibiting caspase-1 activation (Figures 7 and 8).

In summary, our study using *in vivo* and *in vitro* models suggested that caspase-1 is responsible for the pathogenesis of I/R-induced lung injury as a consequence of mediating endothelial pyroptosis and causing barrier dysfunction; VX765 was shown to mitigate these phenomena (Figure 9). Our findings supported VX765 as a new therapeutic approach to limit LIRI and ultimately prevent acute lung injury.

## Data Availability

The datasets used and/or analyzed during the current study are available on reasonable request from the corresponding author.
